# The effect of solvent on Al-doped ZnO thin films deposited *via* aerosol assisted CVD

**DOI:** 10.1039/c8ra06417b

**Published:** 2018-09-25

**Authors:** Dominic B. Potter, Ivan P. Parkin, Claire J. Carmalt

**Affiliations:** Materials Chemistry Centre, Department of Chemistry, University College London 20 Gordon Street London WC1H 0AJ UK c.j.carmalt@ucl.ac.uk

## Abstract

Aluminium-doped zinc oxide (AZO) thin films were deposited *via* aerosol assisted chemical vapour deposition (AACVD) from zinc acetylacetonate and aluminium chloride at 450 °C. The precursor solutions consisted of methanol in a mixture with one other secondary solvent, including toluene, tetrahydrofuran, *n*-hexane, cyclohexane, and ethyl acetate. The crystal structures, elemental compositions and surface morphologies of the resulting AZO films were determined, as well as the optoelectronic properties. It was found that the more polar solvents enhanced growth in the (002) plane of the wurtzite crystal structure, and that solutions with low viscosities resulted in superior grain growth. The film deposited from a solution consisting of methanol and ethyl acetate displayed the lowest visible transmittance, due to carbon contamination. However, it also exhibited 60% lower resistivity, in comparison to the film deposited using methanol only. This suggests that optoelectronic properties can be tuned for specific photovoltaic devices.

## Abstract 2

Aluminium-doped zinc oxide (AZO) thin films were deposited *via* aerosol assisted chemical vapour deposition (AACVD) from zinc acetylacetonate at 450 °C.

## Graphical abstract

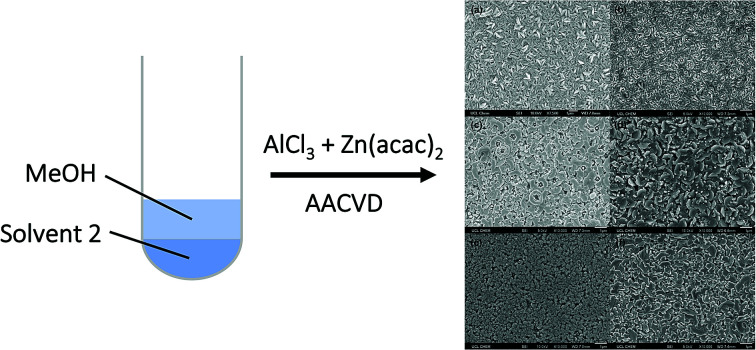

## 1 Introduction

Aerosol assisted chemical vapour deposition (AACVD) is a technique used to deposit thin film coatings.^1^ It is a variation on traditional atmospheric pressure chemical vapour deposition ((AP)CVD), with several key advantages. In APCVD, the precursors are heated in a bubbler to vaporise them, before being transported to a heated substrate *via* a carrier gas, where they react and deposit as a thin film. For this reason, precursors for APCVD must be highly volatile. Conversely, in AACVD, a precursor solution is made up in a suitable solvent, from which an aerosol mist is generated. This mist is transported to the heated substrate, where the solvent evaporates, and the precursors are able to react and deposit. Hence, AACVD has the benefit of opening up a wide range of safe, easy to handle, non-volatile precursors, that would otherwise not be suitable for traditional deposition techniques.^2^

Another advantage of AACVD is that doping is easily achievable, since the stoichiometric ratio of dopant precursors to film precursors in solution can be closely related to the stoichiometric ratio in the resultant film.^3^ By comparison, doping films using APCVD requires precise control over gas flow rates, which can be unreliable, and difficult to reproduce.

There are also cost benefits of using AACVD. In APCVD, the bubbler containing the precursors must be heated to vaporise them. In addition, the piping leading from the bubbler to the substrate must also be heated to prevent condensation of the precursors. In AACVD, however, only the substrate needs to be heated, and hence it is a more energy efficient technique. In addition, AACVD can be performed in an open atmosphere, and a complicated reactor design is not necessary.^4^

Finally, the morphology of the films deposited *via* AACVD can vary, depending on the solvent used to make up the precursor solution.^5^ This means that morphology-related properties can be tuned for particular applications. For example, transparent conducting oxide (TCO) applications generally require large, well-connected grains, to minimise grain boundary scattering and improve transport properties. Some solar cell applications require a high surface roughness, to increase the scattering of incoming light, and to enhance the probability of photon absorption. For films which have been sputtered, a post-deposition etching process must be performed to increase the texture of the film.^6^ With AACVD, this step can be avoided, which adds to the cost-effective nature of this technique. Furthermore, recent work by Powell *et al.* has demonstrated that AACVD has the potential to be scaled up for industrial depositions.^3^

TCOs are a class of semiconductor material, which combine optical transparency with electrical conductivity. This grants them a wide range of applications in optoelectronic devices, such as solar cells, touch screens, light emitting diodes (LEDs) and liquid crystal displays (LCDs).^7^ Currently, the most common industrial TCO materials are fluorine-doped tin oxide (FTO) and tin-doped indium oxide (ITO). However, due to the relatively high and unstable price of raw indium and tin, cheaper alternatives are highly sought after.^8^ One of the main substitute TCO materials that has emerged is aluminium-doped zinc oxide (AZO), due to the inexpensive precursors, the wide band gap of ZnO (*ca.* 3.37 eV at room temperature), and the n-type conductivity.^9^ AZO thin films have been prepared previously by a variety of techniques, including magnetron sputtering,^10^ atomic layer deposition (ALD),^11^ molecular beam epitaxy (MBE),^12^ pulsed laser deposition (PLD),^13^ spray pyrolysis,^14^ APCVD,^15^ and AACVD.^16^

In this work, AZO thin films were deposited *via* AACVD. The effect of varying the solvent used to make up the precursor solution was investigated.

## 2 Experimental

### I Film synthesis

AACVD depositions were performed as detailed in previous work.^17–19^ Nitrogen (99.99%, BOC, Surrey, UK) was used as the carrier gas. All chemicals were obtained from Sigma Aldrich (Dorset, UK), and were used as purchased, without further purification.

A typical precursor solution was made up in a glass bubbler by dissolving zinc acetylacetonate [Zn(acac)_2_] (0.50 g, 1.90 mmol) in a mixture of methanol (MeOH) (10 mL) and another solvent (10 mL) (Table 1). Methanol was used in each case due to the lower solubility of Zn(acac)_2_ in other solvents.

**Table 1:** Solvent mixtures used for AACVD depositions of AZO. The polarity, viscosity, and boiling point values were obtained from the literature^20^

Solvent 1 (10 mL)	Solvent 2 (10 mL)	Polarity of solvent 2	Viscosity of solvent 2/cP	Boiling point of solvent 2/°C
Methanol	Methanol	76.2	0.60	64
Methanol	Toluene	9.9	0.59	110.6
Methanol	Tetrahydrofuran (THF)	21.0	0.55	66
Methanol	*n*-Hexane	0.9	0.31	69
Methanol	Cyclohexane	0.6	0.98	81
Methanol	Ethyl acetate	23.0	0.46	77

Acetic acid (1 mL) was added to aid the solubility and prevent hydrolysis of the Zn(acac)_2_. Aluminium chloride [AlCl_3_] was added, so that there was 10 mol% aluminium in solution, relative to the amount of zinc. This was done because previous work has shown that 10 mol% AZO deposited *via* AACVD had superior electrical conductivities, compared to other dopant concentrations.^17^

The substrate used was a 3.2 mm thick float glass plate (Pilkington Technology Management Limited, Lancashire, UK), which was precoated with a 50 nm thick SiO_2_ barrier layer. This was necessary to prevent leeching of ions between the substrate and the film. The substrate was cut to an area of 10 × 4 cm^2^. It was washed with soap and water, then rinsed with isopropyl alcohol and acetone, before being loaded into the reactor, where it was placed on top of a carbon heater block, which maintained the deposition temperature at 450 °C. A top plate was suspended horizontally, approximately 8 mm above the substrate, to maintain laminar flow of the aerosol.

When the precursor solids were dissolved, the bubbler was placed into a ‘Liquifog’ piezo ultrasonic atomiser from Johnson Matthey, which uses an operating frequency of 1.6 MHz to generate an aerosol. Nitrogen gas at a flow rate of 1 L min^−1^ was used to transport the aerosol over the heated substrate. The reactor exhaust was vented into a fume hood. When the precursor solution and the associated aerosol mist had been completely emptied from the bubbler, the reactor was switched off and allowed to cool to room temperature naturally under a continuous flow of nitrogen gas. The samples were handled and stored in air.

### II Characterisation techniques

X-ray diffraction (XRD) patterns were recorded using a Bruker D8 Discover X-ray diffractometer, which used monochromatic Cu Kα_1_ and Kα_2_ radiation of wavelengths 1.54056 and 1.54439 Å respectively, emitted in an intensity ratio of 2 : 1, with a voltage of 40 kV and a current of 40 mA. The incident beam angle was in a grazing setup at 1°, and data was collected between 10° and 66° 2*θ* with a step size of 0.05° at 2 s per step. X-ray photoelectron spectroscopy (XPS) was performed using a Thermo Scientific K-alpha spectrometer with monochromated Kα radiation, a dual beam charge compensation system and constant pass energy of 50 eV, with a spot size of 400 μm. High resolution scans were performed of the carbon 1s, zinc 2p, oxygen 2p, and aluminium 2p regions. Scans were performed at the film surfaces, and in the bulks of the films after etching for 200 s. Data was fitted using CasaXPS software. Scanning electron microscope (SEM) images were obtained using a JEOL JSM-6301F SEM at an acceleration voltage of 5 kV. Film thicknesses were determined using side-on SEM. Ultraviolet/visible/near infrared (UV/vis/NIR) spectroscopy was done using a Perkin Elmer Lambda 950 UV/Vis/NIR Spectrophotometer in both transmission and in diffuse reflectance mode. Room temperature Hall effect measurements were performed on an Ecopia HMS-3000, which utilises the van der Pauw method. Measurements were taken using a 0.58 T permanent magnet and a current of 1 mA.

## 3 Results and discussion

### I Film synthesis

Methanol (MeOH) has one of the highest polarities of any organic solvent. Many of the solvents used for this study had a relatively low polarity (Table 1), which led to partial immiscibility of some of the solvents with MeOH. For this reason, a phase separation was often observed in the precursor solution prior to deposition. However, this did not appear to affect the deposition, as the process of generating the aerosol using ultrasonic vibrations was enough to continually mix the solution, to avoid any inhomogeneity in the solution. In each case, the entire precursor solution was transported to the reactor, without any solvent left behind. AACVD was successfully used to deposit AZO thin films on glass substrates using a variety of solvent mixtures to make up the precursor solution. Each deposition was repeated at least three times, and no significant discrepancies were observable between repetitions, indicating the reproducibility of the technique.

All of the films were of high quality, and adhered well to the glass substrates, passing the Scotch tape test. The films were visibly transparent, and displayed coloured interference patterns when observed at an angle. These patterns indicate that the film thickness in some regions was similar to that of visible light.^21^ The patterns occur due to interference between photons that reflect from the film–air boundary, and photons that reflect from the substrate–film boundary; this demonstrates the variation in thickness across the film, due to the side-on deposition technique.^22^ The highest quality regions of the films were those located nearest to the baffle inlet, so these regions were characterised. The films were handled and stored in air, and showed no degradation in properties after 12 months.

### II Crystal structure

**Fig. 1:**
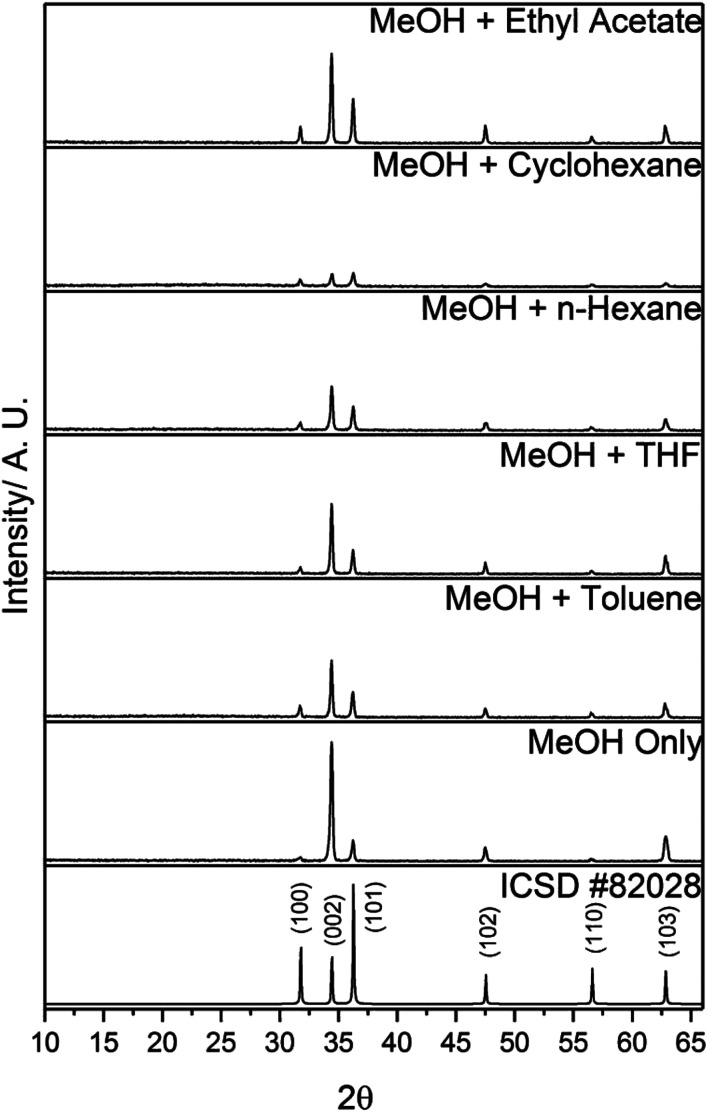
XRD patterns of 10 mol% AZO films deposited *via* AACVD using different solvents. A diffraction pattern of ZnO from ICSD #82028 is also included for reference.

All of the as-deposited films consisted of pure-phase wurtzite ZnO (Fig. 1). No crystalline Al_2_O_3_ phases were observable by XRD. The preferred orientation of the films remained in the (002) plane, regardless of the solvents used. Preferred orientation is commonly observed in thin films and is due to the strain experienced during growth. In ZnO, the (002) plane has the lowest surface energy, and hence preferential growth is usually observed in this direction.^23,24^

The crystallite diameters of the films were estimated using the Scherrer equation:
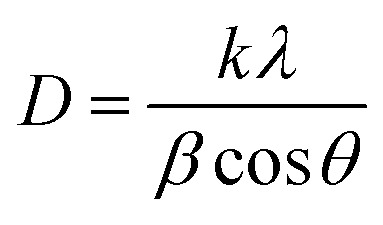
where *D* is the crystallite size, *k* is the Scherrer constant (taken to be 0.9), *λ* is the wavelength of the incident X-rays, *β* is the full-width at half maximum value in radians, and *θ* is the Bragg diffraction angle in radians.^25^ The calculations were performed using the (002) peaks and are presented in Table 2.

**Table 2:** Structural properties of AZO film deposited *via* AACVD using different solvents. The film thicknesses were determined using side-on SEM

Solvent	Preferred orientation	Texture coefficient (002)	Crystallite diameter/nm	Film thickness/μm
MeOH	(002)	3.68	72	1.2
MeOH and toluene	(002)	2.83	92	1.0
MeOH and THF	(002)	3.07	85	1.0
MeOH and *n*-hexane	(002)	2.76	76	0.8
MeOH and cyclohexane	(002)	1.44	62	0.4
MeOH and ethyl acetate	(002)	2.88	85	1.2

The variation in crystallinity indicates that the solvent had a role in crystal growth. In most cases, the addition of a secondary solvent increased the crystallite diameter, and thus improved crystal growth. As the solvent is generally thought to evaporate as the droplets approach the heated substrate, the solvent influence must occur prior to this. Traditionally, it is believed that during a typical AACVD deposition, the aerosol droplet evaporates as it approaches the heated substrate. This leaves the precursors as gaseous molecules which are free to diffuse to the substrate and react heterogeneously to form a solid film. This deposition model suggests that the aerosol is solely used as a transport mechanism, and that the solvent does not have an effect over film growth. However, considering the variation that was observed in the films when different solvents were used, it is more likely that the precursors were initially reacting within the solvent to form clusters and crystallites. After the solvent evaporates, the clusters then diffuse to the substrate and adsorb. The nature of these clusters dictates the nucleation and growth of the film. Hence, varying the solvent can influence the growth of these clusters, which can lead to a variety of film structures. The scheme below shows a general reaction for the AZO film growth:Zn(acac)_2_ + AlCl_3_ → Al_*x*_Zn_1−*x*_O_(cluster)_ → Al_*x*_Zn_1−*x*_O_(film)_

The polarities of the solvents can be related to variations in the growth of ZnO crystallites. Wurtzite ZnO is a polar crystal structure, with alternating layers of Zn^2+^ and O^2−^ stacked along the *c*-axis.^26^ Hence, increasing the polarity of the solvent will enhance growth in the (002) direction, due to strong interactions between the polar (002) surface and the polar solvent, which is likely to reduce the surface energy.^26,27^ This trend was observed in the XRD patterns (Fig. 1), and was quantified by determining the texture coefficients of the films. The texture coefficient is a measure of the degree of preferred orientation, and is calculated from the following equation:
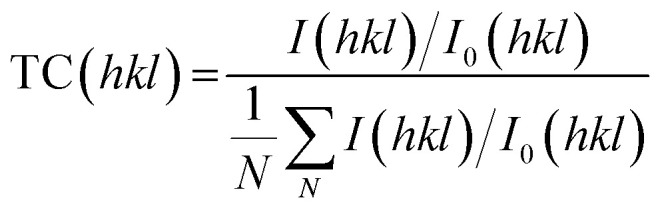
where TC is the texture coefficient of the (*hkl*) plane, *I* is the measured intensity, *I*_0_ is the standard intensity of a corresponding powder pattern, and *N* is the number of reflections.^28^ The TC(002) values are given in Table 2. Deviation of the texture coefficient from unity implies a higher degree of preferred orientation. The general trend observed is that the use of a higher polarity solvent led to the growth of a film which was more textured in the (002) direction. This trend has been observed previously. Yang *et al.* deposited ZnO thin films *via* the chemical bath deposition (CBD) method, using different solvents.^27^ They observed an increase in preferred orientation in the (002) direction when more polar solvents were used. Tomakin *et al.* observed the same phenomenon for their ZnO thin films deposited by spray pyrolysis.^29^ However, they also related the increase in preferred orientation to the higher solubility of their zinc chloride precursor in the more polar solvent, which may also be a contributing factor.

The strain within the films was estimated using the Williamson–Hall method, assuming a uniform deformation model.^30^ All of the films exhibited negative strain, due to the incorporation of Al^3+^ which led to a reduction in unit cell volume. This can be explained by the relatively small ionic radius of Al^3+^ (0.53 Å) compared to Zn^2+^ (0.74 Å).^31^ In general, there was not a significant trend in terms of the amount of strain experienced. However, the film which exhibited the least strain was the film deposited using MeOH only. This may be a result of the interaction between the highly polar solvent and the polar crystal faces, which improved crystal growth and reduced lattice strain.

Since MeOH is one of the most polar organic solvents,^20^ using a secondary solvent with a low polarity led to partial immiscibility, and a phase separation. However, as stated above, this did not appear to affect the deposition. The most nonpolar solvent used in this work was cyclohexane. The film deposited using this solvent possessed the lowest intensity peaks in its XRD data (Fig. 1), the smallest crystallite diameter (Table 2), and the smallest grain diameter (Fig. 4). This suggests that the relatively low polarity of the solution led to poor film growth. This is reflected in the film thicknesses (Table 2), whereby the films deposited with the more polar solvents were thicker than the films deposited with the less polar solvents.

### III Elemental analysis

XPS was used to ascertain the composition of the films. Adventitious carbon was observed both at the surfaces and within the bulks of the films, and was used to calibrate the data with a binding energy of 285.0 eV for the carbon 1s peaks. Carbon is often observed in the XPS data of thin films. This is partly due to the inefficient thermal degradation of the Zn(acac)_2_ precursor, in the low oxygen environment within the reactor. The carbon peaks varied in intensity for the films deposited using different solvents, which suggests that the solvent was also contributing towards carbon contamination. The film with the highest concentration of carbon was the film deposited using ethyl acetate. The increased levels of carbon contamination led to a decrease in visible transmittance, as discussed below.

**Fig. 2:**
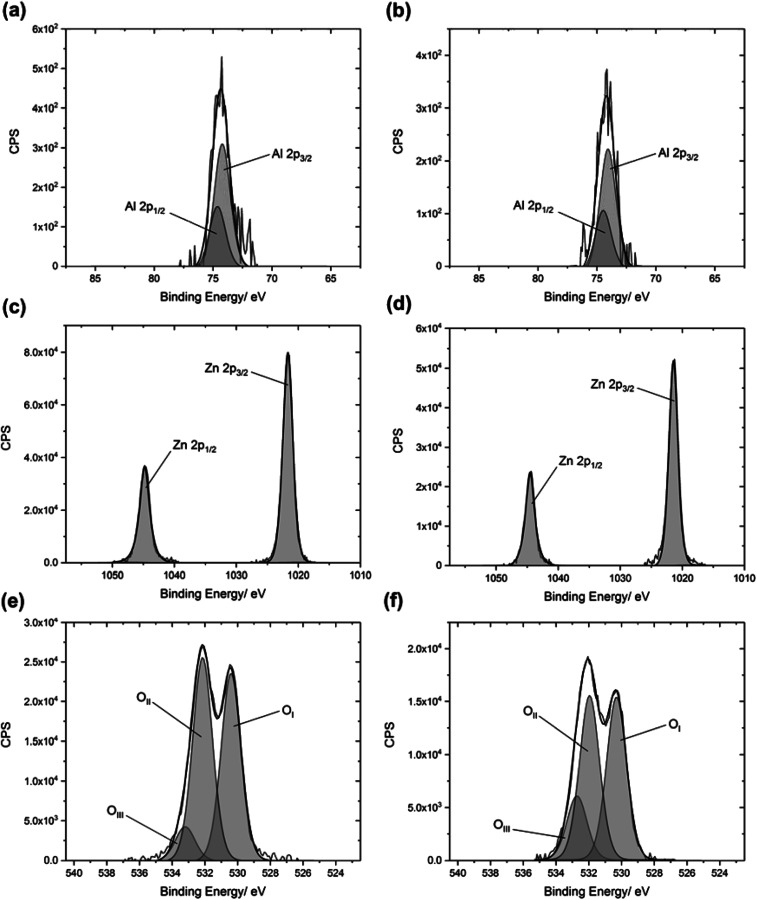
Typical surface XPS spectra for the AZO thin films, showing the Al 2p peaks from the films deposited using (a) MeOH and ethyl acetate and (b) MeOH and THF; the Zn 2p peaks from the films deposited using (c) MeOH and ethyl acetate and (d) MeOH and THF; the O 2p peaks from the films deposited using (e) MeOH and ethyl acetate and (f) MeOH and THF. “CPS” refers to counts per second.

Typical XPS spectra are shown in Fig. 2. The binding energies of the zinc 2p_3/2_ and 2p_1/2_ peaks in each film were found to be 1021.3 eV (±0.2 eV) and 1044.4 eV (±0.2 eV), respectively, which can be attributed to the Zn^2+^ in ZnO. The oxygen 2p peaks consisted of three environments: O_I_ at 530.4 eV (±0.2 eV), O_II_ at 532.1 eV (±0.2 eV), and O_III_ at 533.2 eV (±0.2 eV). O_I_ is due to the presence of the O^2−^ ions in ZnO. O_II_ is caused by O^2−^ ions which are located in oxygen deficient regions of the lattice, and thus relates to the number of oxygen vacancies present. O_III_ arises due to surface-bound impurities.^19^ The relative intensities of the O_II_ and O_III_ peaks decreased after etching into the film, indicating a decrease in the concentration of oxygen vacancies within the bulk of the film, and a loss of surface-bound impurities.

**Fig. 3:**
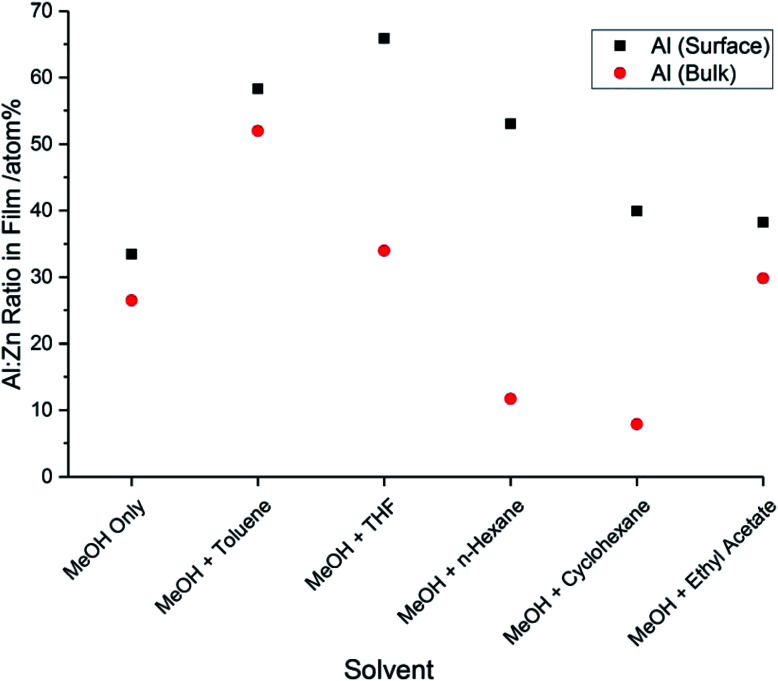
Al : Zn ratios at the surfaces and in the bulks of the AZO films deposited *via* AACVD using different solvents, as determined by XPS.

The binding energies of the aluminium 2p_3/2_ and 2p_1/2_ peaks in each film were found to be 74.2 eV (±0.2 eV) and 74.6 eV (±0.2 eV), respectively. These values can be assigned to Al^3+^.^32^ The characteristic peak for metallic Al at 72.6 eV was not observed.^33^ For each film, the concentration of aluminium was higher at the surface compared to the concentration within the bulk (Fig. 3). This surface segregation was due to the formation of Al_2_O_3_ at the grain boundaries, possibly as a result of aluminium reaching its solubility limit in the ZnO film.^34^ This Al_2_O_3_ is likely to be amorphous and low in concentration, so it was not detectable by XRD.

The concentrations of Al^3+^ at the surfaces of the films and within the bulks were determined from the ratios between the aluminium 2p peaks and the zinc 2p peaks. It was found that the concentration of Al^3+^ varied with the solvent used to make up the precursor solution. The use of MeOH and toluene resulted in an increase in the incorporation of Al^3+^, both at the surface and within the bulk. When THF was used, the concentration of Al^3+^ within the bulk was similar to when MeOH was the only solvent, but the concentration at the surface increased significantly. This indicates that there was an increase in the concentration of Al_2_O_3_ segregated to the grain boundaries. Similarly, when *n*-hexane or cyclohexane were used, the amount of Al^3+^ incorporated into the film bulks decreased, whereas the amount segregated at the surface increased. As Al_2_O_3_ is an insulating phase, it will cause a deterioration in the electrical properties of these films. The low concentration of Al^3+^ in the bulks of the films deposited using *n*-hexane and cyclohexane can be explained by the fact that AlCl_3_ has a low solubility in these solvents, due to their nonpolar nature. The low concentration of Al^3+^ in these films will result in a low carrier concentration, which will further reduce the conductivity. The film deposited using MeOH and ethyl acetate had very similar Al^3+^ concentrations, when compared to the film deposited using MeOH only.

### IV Surface morphology

**Fig. 4:**
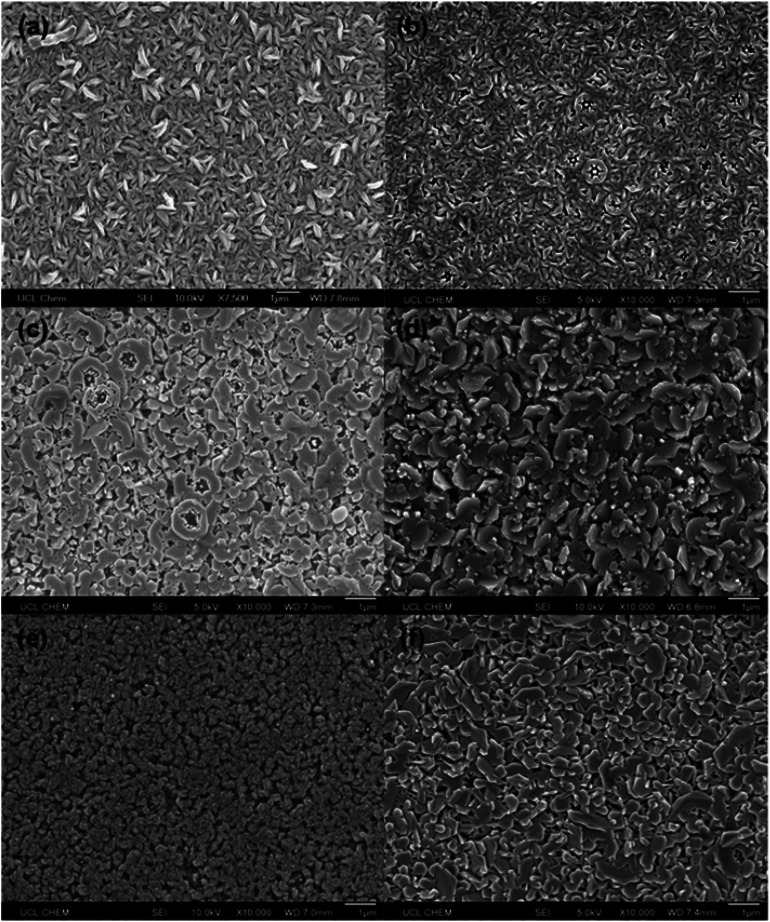
SEM images of 10 mol% AZO deposited *via* AACVD using (a) MeOH only, (b) MeOH and toluene, (c) MeOH and tetrahydrofuran, (d) MeOH and *n*-hexane, (e) MeOH and cyclohexane, and (f) MeOH and ethyl acetate as the solvents used to make up the precursor solution.

SEM imaging revealed a variation in surface morphologies for the films deposited using different solvents (Fig. 4). Overall, the films were polycrystalline, with grain diameters up to 1 μm. This is much larger than the crystallite diameters calculated in Table 2. The discrepancy can be explained by the fact that the grains themselves are made up of much smaller crystallites which are in the same orientation.^35^ In some of the films, etched hexagonal grains could be seen, which is commonly observed for ZnO thin films, due to the hexagonal wurtzite crystal structure. However, the majority of the grains were more randomly shaped.

Large, well-connected grains are preferable for ZnO-based TCO applications, as they will minimise grain boundary scattering.^19^ The films deposited using MeOH and THF, MeOH and *n*-hexane, and MeOH and ethyl acetate had the largest grains. Notably, THF, *n*-hexane, and ethyl acetate all have lower viscosities than MeOH (Table 1), suggesting that a solution with a lower viscosity resulted in improved grain growth. The reason for this could be that the aerosol can be generated more easily and consistently from a lower viscosity solution. Wang *et al.* measured the droplet sizes of different aerosols generated using an ultrasonic nebulizer.^36^ They found that decreasing the viscosity of the solution led to a decrease in the mean droplet size, and that this led to an increased spray volume rate. Hence, with a lower viscosity solution, there will be a more rapid, continuous supply of precursors to the substrate, resulting in improved growth mechanics. This is in contrast to the films deposited using MeOH only, MeOH and toluene, and MeOH and cyclohexane, which had smaller grains. Cyclohexane is not typically used as the sole precursor solvent for AACVD due to its high viscosity (Table 1), which makes it difficult to generate an aerosol. The film deposited using MeOH and toluene was very similar to the film deposited using MeOH only, possibly because the two solvents have comparable viscosities, which means the aerosols generated from the two precursor solutions would be very similar.

### V Optical properties

**Fig. 5:**
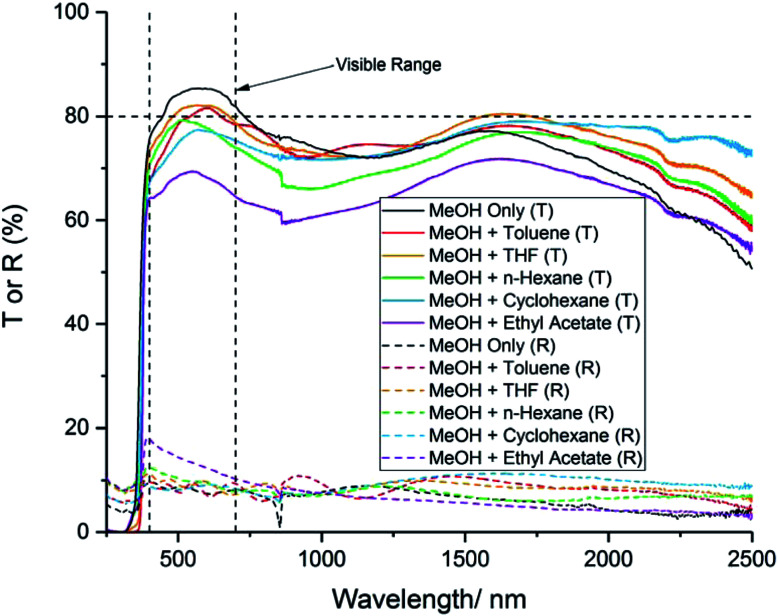
Transmission–reflectance spectra for 10 mol% AZO deposited *via* AACVD using different solvents. “*T* or *R*” refers to transmission or reflectance.

For TCO applications, the average transmittance across the visible part of the spectrum (400–700 nm) should be >80%. The transmission–reflectance plots for the films generally showed high transmittance across the wavelengths scanned (Fig. 5). However, all of the films that used a solvent mixture showed a lower transmittance in the visible part of the spectrum, compared to the film deposited using MeOH only (Table 3). This could be due to an increase in carbon contamination from the different solvents, which is known to cause a visible darkening in thin films.^37^ It is worth noting that MeOH has the lowest boiling point of any of the solvents used for this study (Table 1). This could indicate that MeOH is more likely to evaporate prior to reaching the substrate surface, compared to the other solvents. Hence, it is possible that MeOH will be transported away by the carrier gas, and passed through the exhaust, before it has a chance to contaminate the film with carbon. Hassan *et al.* found that, for their indium oxide films deposited *via* AACVD, the use of toluene as the solvent for their precursor solution resulted in an increase in carbon contamination, compared to the films deposited using a MeOH solution.^38^ Similarly, Kafizas *et al.* have previously reported that ethyl acetate can cause significant carbon contamination in films deposited *via* APCVD.^22,39^ This explains why the film deposited using ethyl acetate had the highest carbon concentration (as revealed by XPS), and displayed the lowest visible transmittance.

**Table 3:** Optoelectronic properties of 10 mol% AZO films deposited *via* AACVD using different solvents. *T*_λ400–700_, average transmittance in the visible part of the spectrum (400–700 nm); *E*_g_, band gap; *ρ*, resistivity; *n*, carrier concentration; *μ*, mobility

Solvent	*T*_λ400–700_/%	*E*_g_/eV	*ρ* × 10^−2^/Ω cm	*n* × 10^19^/cm^−3^	*μ*/cm^2^ V^−1^ s^−1^
MeOH	83	3.25	0.5	14.0	9.0
MeOH and toluene	78	3.25	27.2	7.1	0.3
MeOH and THF	80	3.25	44.2	5.3	0.3
MeOH and *n*-hexane	77	3.25	N/A	N/A	N/A
MeOH and cyclohexane	75	3.26	N/A	N/A	N/A
MeOH and ethyl acetate	67	3.24	0.2	9.7	13.3

The reflectance of the films did not vary significantly with precursor solvent, and remained low (∼5–15%) across the wavelengths scanned. This could be because the carrier concentrations and carrier mobilities were not high enough to result in plasma resonance in the near IR region.^40^ The low reflectance of the films indicates that they would not be suitable for low-emissivity coatings, which require a high reflectance in the IR range.^41^

The band gaps of the films were calculated from the transmission–reflectance data, using the Tauc method.^42^ The values are presented in Table 3. No significant difference in the band gap was observed when different solvents were used to make up the precursor solution. The band gaps are similar in value to 10 mol% AZO films deposited previously *via* AACVD.^17,43^

### VI Electrical properties

The resistivity, carrier concentration and mobility for each film was determined using the van der Pauw technique, and the results are summarised in Table 3. The films deposited using MeOH and *n*-hexane, and MeOH and cyclohexane were both too resistive to determine the electrical properties by this method. The rest of the films were all n-type semiconductors, which is expected for AZO.^44^

The conductivity of the films was due to the incorporation of Al^3+^ into the lattice, which introduced shallow donor levels beneath the conduction band minimum (CBM). Very low concentrations of nitrogen from the carrier gas may have also been incorporated into the lattice, although this is not thought to have affected the electrical properties. In ZnO, molecular nitrogen introduces a state which acts as a hole trap.^45^ This would be problematic for p-type ZnO, but the effect would be negligible for the n-type ZnO films in this work.

The resistivity values varied by two orders of magnitude for the other films, indicating that the solvent used to make up the precursor solution had a profound effect on the functional properties of the films. The addition of toluene or THF to the precursor solution resulted in a decrease in carrier mobility by over an order of magnitude, compared to when only MeOH was used, which resulted in a significant increase in resistivity (Table 3). The inferior electrical properties of these films may be due to the poorer crystal quality, as indicated by the relatively low intensity peaks in the XRD pattern (Fig. 1). Crystallographic defects can hinder carrier mobility, which limit transport properties. In the case of the film deposited using THF, there is the additional factor of the relatively high amounts of Al^3+^ segregated to the surface (Fig. 3), indicating a high concentration of insulating Al_2_O_3_ at grain boundaries. This was also observed for the films deposited using *n*-hexane and cyclohexane, which were too resistive to be able to determine their electrical properties using the van der Pauw technique. As stated above, the low concentration of Al^3+^ in the bulks of these films is due to the low solubility of AlCl_3_ in these solvents. Hence, the grain boundary scattering, coupled with the low concentration of Al^3+^ to supply charge carrying electrons to the conduction band resulted in poor electrical properties for these films. The use of MeOH and ethyl acetate resulted in an improvement in the electron mobility, leading to a 60% reduction in resistivity compared to when only MeOH was used. This was due to the high-quality crystallinity of the film, as shown in its XRD pattern (Fig. 1), and its relatively large, smooth grain structure, which provided good pathways for conducting electrons to travel through (Fig. 4). This is a significant finding, as it is preferable to improve the resistivity of optoelectronic devices by increasing the carrier mobility, rather than increasing the carrier concentration, since a high carrier concentration can cause a deterioration in optical properties.

For most TCO applications, the resistivity should be on the order of 10^−4^ Ω cm. The film deposited using methanol and ethyl acetate is approaching this value, and demonstrates that the electrical properties of films deposited *via* AACVD can be improved simply by varying the precursor solvent. The resistivity value of the film deposited using methanol and ethyl acetate is comparable to previous work in which AZO was deposited *via* AACVD. Kuprenaite *et al.* deposited AZO onto a glass substrate *via* AACVD, at a deposition temperature of 400 °C, and achieved a much poorer resistivity value of 2.83 Ω cm. Both Bhachu *et al.* and Ponja *et al.* deposited AZO thin films onto glass substrates *via* AACVD at 450 °C, and achieved resistivities of 8.35 × 10^−4^ Ω cm and 2.15 × 10^−3^ Ω cm, respectively. However, in each case the precursors used were the highly pyrophoric diethyl zinc and triethyl aluminium, which made the depositions non-trivial and hazardous. Resistivity values on the order of 10^−5^ Ω cm have been obtained in AZO thin films deposited *via* PLD.^46^ However, this technique is expensive, and does not permit the same level of morphological control as depositions performed *via* AACVD.

## 4 Conclusion

AZO thin films were successfully deposited onto glass substrates *via* AACVD, using various methanolic dual solvent mixtures for the precursor solution. The growth of the film was shown to be highly dependent on the solvent. The more polar solvents enhanced growth in the polar (002) plane in the wurtzite crystal structure. The less polar solvents resulted in films with a lower bulk concentration of Al^3+^, due to its lower solubility in solution. In addition, a precursor solution with a lower viscosity resulted in superior grain growth. This can be explained by the fact that the aerosol could be generated more consistently, which led to a faster, more continuous supply of precursor to the growing film. The film deposited using MeOH and ethyl acetate displayed the lowest visible transmittance, due to a high amount of carbon contamination. However, this film also exhibited a 60% reduction in resistivity, in comparison to the film deposited using MeOH only, due to the improvement in crystal structure and morphology. This work has demonstrated how the surface morphology and optoelectronic properties of TCO materials deposited *via* AACVD can be tuned for various photovoltaic devices, simply by varying the solvent used for the precursor solution.

## Conflicts of interest

There are no conflicts to declare.

## Supplementary Material
